# Impact of cone-beam computed tomography for the identification and management of an oral portal of entry in patients with infective endocarditis. A Delphi study

**DOI:** 10.4317/medoral.24885

**Published:** 2021-09-25

**Authors:** Alexandra Cloitre, Emilie Hascoët, Bernard Iung, Xavier Duval, Philippe Lesclous

**Affiliations:** 1 DDS, PhD, INSERM, UMR-S 1229, RMeS; Université de Nantes, CHU de Nantes, France; 2 DDS, INSERM, UMR-S 1229, RMeS; Université de Nantes, CHU de Nantes, France; 3 MD, PhD, Hôpital Universitaire Bichat, Université de Paris, France; 4 MD, PhD, INSERM CIC 1425, AP-HP, Hôpital Universitaire Bichat; INSERM U1137 IAME; Université de Paris, France

## Abstract

**Background:**

Infective endocarditis (IE) is a rare and life-threatening disease. Cutaneous portal of entry (POE) is predominant for IE, but an oral POE is the second most frequent source. Thus looking for and treating an oral POE in IE patients is of critical importance in order to reduce the risk of IE relapse or recurrence. The objectives of this study were: 1) To reach a consensus on decision-making following the detection of an oral POE on cone-beam computed tomography (CBCT) while they were not identified using the current recommended approach in IE patients (oral examination and orthopantomogram: OPT). 2) To determine whether this consensus differs when regarding the microbiology of IE.

**Material and Methods:**

Twenty oral or maxillofacial surgeons participated to this Delphi study. The questionnaire was based on five radiological cases (OPT and matching CBCT) with two scenarios according to the objectives of detecting oral POE in an IE patient (curative in case of oral causative microorganism, and preventive if not) and different therapeutic approaches (surgical or conservative treatment, no treatment) for each of them. Consensus was defined as an agreement rate of ≥75%.

**Results:**

The response rate was≥85%. After four rounds, consensus was achieved for all proposals. CBCT changed the decision-making of experts in four cases. In one case, the decision was influenced by the IE microbiology toward a more radical approach in case of oral causative microorganism.

**Conclusions:**

In IE patients, CBCT changed markedly the decision-making of experts by eradicating more oral POE than when using OPT. This could reduce the risk of IE relapse and recurrence.

** Key words:**Cone beam computed tomography, orthopantomogram, clinical decision-making, oral infectious foci, infective endocarditis, Delphi study.

## Introduction

Abbreviations: CBCT: Cone beam computer tomography; IE: Infective endocarditis; POE: Portal of entry; OIF: Oral infectious foci; OPT: Orthopantomogram.

Infective endocarditis (IE) is a rare (3–10 cases per 100,000 persons per year) and life-threatening disease with a mortality rate of 30% at 1 year (1). In patients with a previous history of IE, 5 to 10% will have additional episodes of IE (2). According to the European Society of Cardiology, theses patients are among those at highest risk of IE (Table 1) (3). It is well documented that cutaneous portal of entry (POE) is predominant for IE, but an oral POE is identified as the second most frequent source in about 30% of the IE patients (4,5). Thus looking for and treating an oral POE in IE patients is of critical importance in order to reduce the risk of IE relapse or recurrence.

In a recent position paper, a French multidisciplinar task force highlighted general priciples for oral evaluation of patients with heart valve disease including during acute IE (6). Cardiologists and cardiac surgeons are generally the first practitioners involved in the initial evaluation or follow-up of such patients and they should be aware of the detection of an oral POE. For this purpose, a short questionary based on simple items has been proposed for a non oral specialist (Table 2) (6). If relevant, this first-line evaluation should lead to a thorough evaluation by a dentist.

All the current guidelines for dentists promote systematic detection of oral infectious foci (OIF) and elimination of all of them in IE patients (3,7,8). But the modalities for screening and manage OIF are not always consensual and well detailed. Generally, a careful oral examination based upon clincal symptoms and patients’ complaints is first mandatory. This examination is recommended to be systematically completed with a two-dimensional (2D) conventional radiographs (mainly orthopantomogram: OPT) not only to assess OIF with clinical symptoms but also to detect asymptomatic OIF.

**Table 1:**
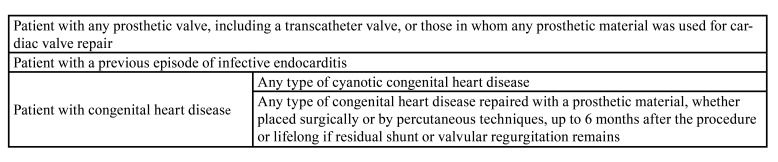
Cardiac conditions at highest risk of infective endocarditis.

**Table 2:**
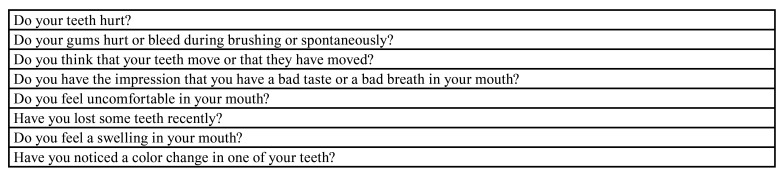
Standard questions to be asked by the non-specialist to patients with valvular heart disease. At least, one positive answer should lead to a consultation with a dentist.

However, this recommended approach appears to be limited. In a recent cross-over study comparing the orodental status of IE patients with an oral causative micro-organism or non-oral causative micro-organism, no difference was recorded (9). Moreover, oral examination coupled with an OPT failed to objectivate an oral POE in approximately 30% of IE patients with an oral causative micro-organism (9). This suggests that oral examination coupled with OPT is not sensitive enough to detect asymptomatic OIF in IE patients, thus keeping them at high risk of IE relapse or reinfection.

For this reason, the French multidisciplinar task force for the evaluation and management of oral status in patients with valvular disease suggested the systematic use of three-dimensional (3D) cone beam computed tomography (CBCT) for IE patients in order to improve the detection of asymptomatic OIF (6). Indeed, CBCT has superior diagnostic accuracy than conventional radiographs in detecting OIF (sensitivity 0.95 vs. 0.56 and specificity 0.88 vs. 0.78, respectively) (10).

However, while there is consensus about eradicating OIF detected on OPT in IE patients, regarding this issue when OIF are only detected using CBCT is an ongoing professional debate (3,6). The use of CBCT would be ethically (additional radiation) and economically justifiable if the additional OIF detected with this imaging modality would have a significant impact on the management of such patients.

To analyze the impact of CBCT in IE patients, we performed a Delphi study whose aims were 1) to reach professional consensus on decision-making following the discovery of OIF on CBCT while they were not identified using the current recommended approach in IE patients (an oral examination coupled with a 2D conventional radiograph: OPT), and 2) to determine whether this consensus differs when regarding the microbiology of IE. The Delphi process is a valuable source of evidence in health-care research (11).

## Material and Methods

1. Rationale for the Delphi technique

Use of the Delphi technique is appropriate for achieving expert consensus on an issue of uncertainty and no quantifiable measure of outcome in clinical practice (12,13). In this study, it was the decision-making following OIF detection in IE patients using CBCT as an assistant tool of conventional OPT. The present study was conducted and reported using the CREDES guidelines (12).

2. Panel selection

A total of 20 certified oral or maxillofacial surgeons scattered all over the French country were selected to participate in this Delphi study. The main eligibility criterion was experience of more than 10 years regarding OIF detection and management. A hospital-based practice was mandatory in this study dedicated to IE patients in hospital. The panel members are listed in the Acknowledgments section.

3. Delphi procedure

Preparatory phase: Five conditions of OIF according to the guidelines of the French Society of Oral Surgery were selected, All of them were only detected on CBCT images but not on OPT and not revealed by clinical examination performed by the 2 supervisors of this study, (AC and PL): 1) endodontically treated first right mandibular molar with apical periodontitis, 2) jaw cyst in an edentulous area of the left mandible, 3) incomplete endodontic treatment of the first right upper premolar without apical periodontitis, 4) small but deep decay of the upper left second premolar, and 5) jaw cyst associated with an impacted upper left premolar. The Delphi questionnaire was constructed and implemented in the online survey tool LimeSurvey®. It was previously tested by the 2 supervisors of this study, who did not take part in the expert panel.

**Figure 1:**
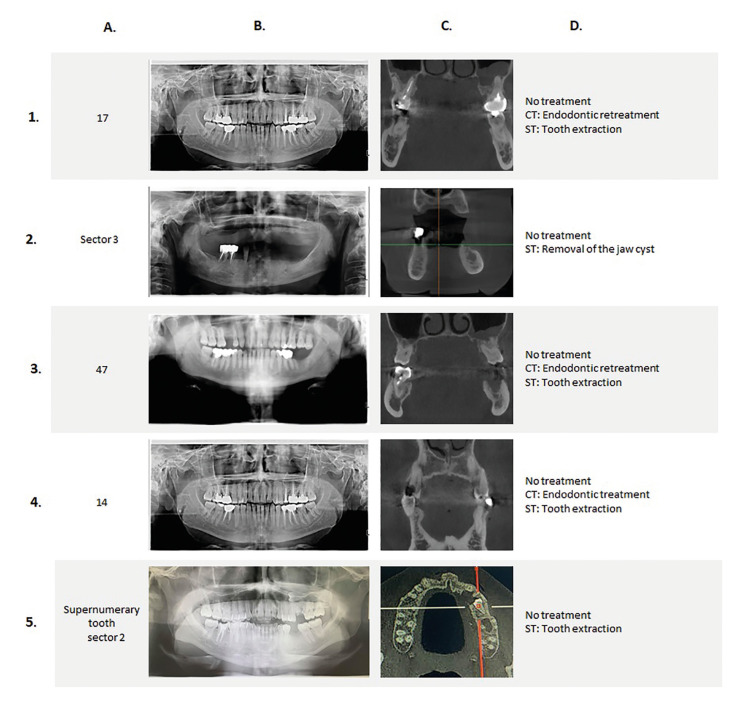
Overview of the Delphi questionnaire. In the five cases (from 1 to 5), in the region of interest (A), orthopantomograms (B) do not show any abnormality whereas abnormalities are discernible in the matching CBCT (C). According to the conditions, two or three therapeutic alternatives were available (D): no treatment, conservative treatment (CT), and surgical treatment (ST).

Questionnaire: The Delphi questionnaire consisted of five radiological cases (OPT and key sections of the matching CBCT) illustrating the five conditions of OIF previously identified (Fig. 1).

In each of these cases, clinical examination coupled with OPT which is the first-line approach recommended in current guidelines, failed to evidence an abnormality whereas OIF was discernible in the matching CBCT. This allowed us to assess the impact of CBCT on the decision-making of experts. For each radiological condition, two scenarios were described according to the objective of detecting OIF in an IE patient: 1) curative objective (in case of oral micro-organism IE) to manage an oral POE of the current IE episode; and 2) preventive objective (in case of non-oral micro-organism IE) to prevent a potentially new IE episode. And for each scenario, three proposals for the management of OIF (surgical treatment, conservative treatment, no treatment) were submitted to the panel of experts. For each proposal, panelists were asked to indicate the extent of their agreement on a 9-point Likert scale (from 1 = strongly disagree to 9 = strongly agree) and free text areas were provided for respondents' comments.

**Figure 2:**
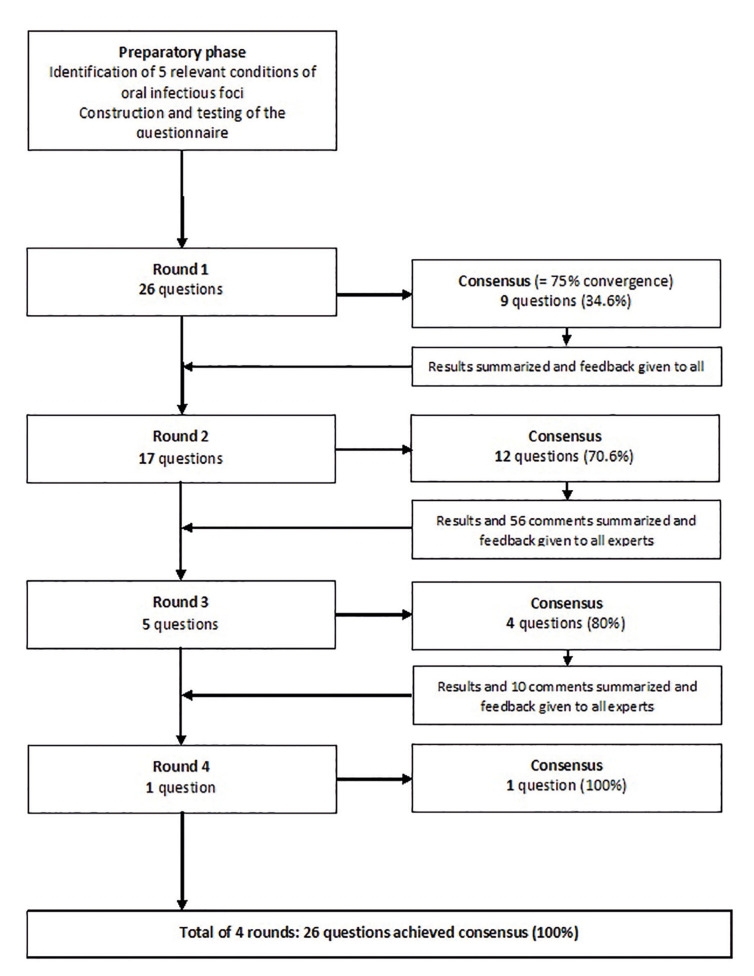
Flowchart of the Delphi process.

Delphi process: A four-round online Delphi study was carried out between February and April 2020. Current guidelines for the prevention of IE were provided to the experts along with the questionnaire (6,7). Reminder e-mails were sent 1 week after the initial invitation of each round. All the experts voted anonymously and independently. The flow chart in Fig. 2 shows the phases and the process of the four consecutive Delphi rounds (Fig. 2).

After the first round, a feedback report presenting the descriptive results was sent to the panelists. Consensual proposals were withdrawn from the questionnaire after each round. Non-consensual proposals were reworded into a new questionnaire, exclusively based on previous comments from experts to clarify the remaining conditions. The same way of proceeding was performed after each round until the ultimate fourth round.

4. Data collection and analysis

Data from fully completed questionnaires were extracted from LimeSurvey® and were imported into Microsoft Excel 2019 (Microsoft Corporation, Issy les Moulineaux, France) for descriptive analysis. The level of agreement of the experts was indicated in two ways: 1) by the mean scores ± SD that they attributed to the proposals on the 9-point Likert scale and 2) by evaluating the consensus reached. This assessment was performed after the scores assigned by the experts were aggregated into three categories (1–3 = disagree, 4–6 = neither agree nor disagree, 7–9 = disagree). Consensus was considered to be reached when at least 75% of the panelists indicated the same category (12). The consensus was indicated in terms of agreement with the proposal, the number (n) and percentage (%) of experts involved, and the number of rounds needed to reach consensus (Table 3).

## Results

1. Panelist characteristics and response rate

Eleven of the participants were male (55%) and 9 were female (45%); the male/female sex ratio was 1.2. Of the 20 panelists selected to participate to this Delphi survey, 17 completed questionnaires in Round 1 and 18 in the other three rounds (response rate of 85% and 90%, respectively).

2. Delphi process

Consensus was reached in Round 1 on 34.6% of the proposals (9/26), and in 70.6% (12/17), 80% (4/5), and 100% (1/1), respectively, of the three following rounds for the remaining proposals (Fig. 2). For each round, comments were made by the experts (56, 10, and one comments for Rounds 2, 3, and 4, respectively). They were used to reword five of the 26 initial proposals.

**Table 3:**
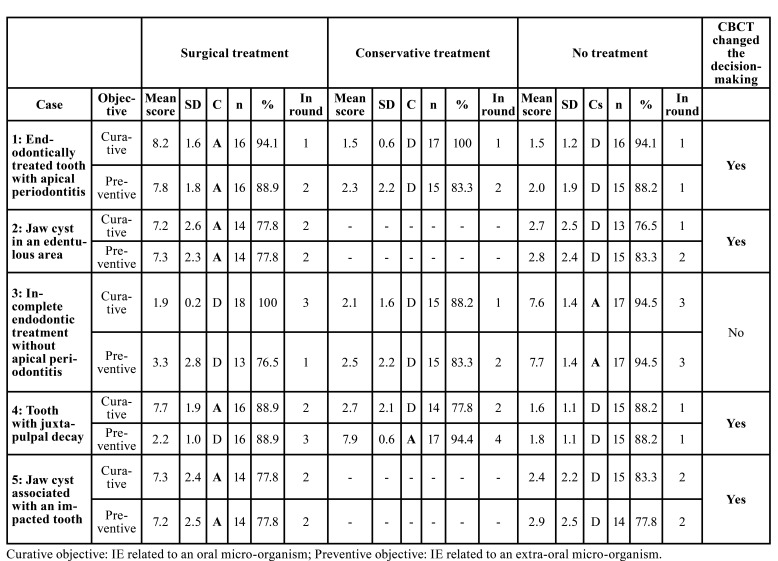
Results of the Delphi survey. The experts' level of agreement is shown as mean scores attributed to the therapeutic proposals on a 9-point scale (from 1 = strongly disagree to 9 = strongly agree) and standard deviations (SD). Consensus achieved was calculated after the scores assigned by the experts were aggregated into three categories (1–3 = disagree, 4–6 = neither agree nor disagree, 7–9 = disagree). Consensus (≥ 75% convergence) (C) is indicated in terms of agreement (A) or disagreement (D) with the therapeutic proposal, the number (n) and percentage (%) of experts involved, and the number of rounds for reaching consensus. The change in expert decision-making after CBCT is indicated for each case (Yes, No).

3. Cases (Table 3)

Case 1: Endodontically treated first right mandibular molar with apical periodontitis.

Consensus was reached on all proposals (surgical or conservative treatment) for a curative objective in Round 1, and in Round 2 for a preventive objective. Consensus on excluding therapeutic abstention was reached in the first round. CBCT influenced the decision-making of the experts in this case.

Case 2: Jaw cyst in an edentulous area of the left mandible

Regarding this case, for both objectives a consensus on surgical management was reached after two rounds. No conservative option was proposed for this case. The consensus to exclude therapeutic abstention also took two rounds to reach. CBCT changed the management of this case by the experts.

Case 3: Incomplete endodontic treatment of the first right upper premolar without apical periodontitis

For this case, consensus on surgical management took three rounds to reach and required a rewording of the proposal after Round 2, while consensus on conservative management was reached after two rounds. Consensus on therapeutic abstention also took three rounds to reach after rewording following the second round. CBCT did not change the attitude of the experts in this case.

Case 4: Small but deep decay of the upper left second premolar

This was the most debaTable case. Consensus on a surgical option took three rounds and for a conservative option four rounds to reach, both after rewording of the proposals. Interestingly, the consensual treatment recommended for a curative objective was surgical treatment, while it was conservative treatment for a preventive objective. Consensus on excluding therapeutic abstention was reached after the first round. CBCT changed the expert management of this case.

Case 5: Jaw cyst associated with an impacted upper left premolar

Consensus was reached on surgical management in the second round, both for preventive or curative objectives. Conservative management was not an available option for this case. Consensus on excluding therapeutic abstention was also reached in the second round. CBCT changed the decision-making of experts in this case.

Taken together, these data indicate that consensus was obtained in Round 2 for all proposals of Cases 1, 2, and 5, in Round 3 for those of Case 3, and in Round 4 for those of Case 4. CBCT changed the decision-making of experts in four of the five cases for a curative objective and in three cases for a preventive objective.

## Discussion

This Delphi study shows that in IE patients, CBCT impacted the decision-making of experts by increasing the number of OIF to eradicate when oral examination and OPT, the currently recommended approach for OIF identification in IE patients, were not conclusive. This finding is of major importance because this could potentially reduce the risk of IE relapse or recurrence. Although the high diagnostic performance of CBCT is well established for OIF detection, this is the first time that an impact on clinicians' decision-making has been evidenced in the crucial context of the treatment and prevention of IE.

In IE patients, the impact of screening and management of OIF is dual since both aim to eradicate a current or a potential POE for IE. Because patients with recurrent IE have a greater need for valve replacement and a higher risk of death, and due to the high sensitivity of CBCT in detecting OIF, a recent position paper of a multidisciplinary task force proposed the systematic use of CBCT in addition to clinical examination for these patients (6).

The five clinical cases presented in the questionnaire illustrate the limitations of a conventional approach coupling oral examination to 2D imaging in detecting asymptomatic OIF that were revealed by CBCT. The low definition of OPT often limits the detection of small lesions of the jaws (Case 1). Larger lesions may also be undetected because their location in the medullary bone is occulted by a consistent cortical bone layer (Case 2). Kinetic blurring (Case 3), superimposition of adjacent anatomical structures, such as a tooth (Case 4) or maxillary sinus (Case 5), or a location in an out-of-section area (Case 1) are other limitations of OPT, which could result in under-detection and therefore under-elimination of OIF.

Some limitations of OPT can be overcome with CBCT. This highly detailed imaging modality is currently considered as the standard 3D technique in dental and maxillofacial radiology (14). There is evidence that CBCT has better diagnostic accuracy than conventional radiographs, i.e., periapical radiographs (considered to be more accurate than OPT) for detecting apical periodontitis, a frequent source of OIF in the general population (10). However, CBCT generally results in higher radiations doses than a single conventional radiograph. A recent meta-analysis has shown that the adult effective doses for CBCT vary widely, from 5 to 1073 µSv, with a mean dose depending on the field of view size from 84 µSv to 212 µSv (15). In comparison, the effective dose from OPT is between 2.7 and 75 µSv (14). According to the ALADA (as low as diagnostically accepTable) principle, the radiation dose for each examination should be justified and optimized to achieve diagnostically suiTable images. To guide clinicians, evidence-based guidelines for the use of CBCT in dental and maxillofacial practice have been published by several institutions such as the European Commission or the American Dental Association (16). These guidelines did not recommend CBCT for screening purposes, but they also did not mention patients with high infectious risk such as IE patients, for whom the expected potential clinical benefits could likely outweigh the risks associated with ionizing radiation exposure such as radiation-induced cancers.

Our Delphi study showed for the first time that CBCT had a significant impact on the decision-making for IE patients. This led experts in four out of five cases to decide for a treatment to eradicate the OIF, mainly through a surgical approach. This is in agreement with previous studies in endodontics and orthodontics showing that CBCT could lead to a more invasive treatment (17-20). The only case for which experts recommended therapeutic abstention was Case 3: incomplete endodontic treatment without apical periodontitis. This may be due to two non-evidence-based factors: the presumed low virulence of this type of OIF ranging 1.6/10 according to the French Society of Oral Surgery and the contraindication of endodontic retreatment in IE patients in some guidelines (5,6).

Importantly, consensus was reached for each proposal in our study. It was obtained in the first two rounds for all proposals of Cases 1, 2, and 5 and in later rounds for those of Cases 3 and 4 with presumably less virulent OIF according to the French society of oral surgery, was and so more debaTable management.

In addition to changes induced in decision-making, another parameter for assessing the relevance of CBCT is the certainty of practitioners in their therapeutic choice. In our study, it should be noted that the mean scores of the therapeutic proposals on a 9-point Likert scale were generally high (range 1.5–3.3 for agreement and 7.2–8.2 for disagreement).

Although CBCT influenced the decision-making of experts in our study by leading to the eradication of more OIF than with OPT, an important remaining question is whether it is appropriate or not to eradicate all the additional OIF detected by CBCT to prevent a new IE episode. To answer this question, the performance of OPT in detecting an oral POE for IE has to be determined first. In a single-center prospective study using conventional radiographs, an oral POE was identified in 29% of IE patients in whom the current episode of IE was presumed to be mainly related to the presence of OIF (87%) and more rarely to invasive dental procedures (12%) (5). Among the IE patients with a non-identified POE, 22% of OIF were caused by oral micro-organisms. This suggests that OPT failed to show OIF that could be discernible with CBCT. In another study, oral examination coupled with an OPT also failed to objectivate an oral POE in approximately 30% of IE patients with an oral causative micro-organism (9).

Another point to be addressed in response to the question is the assessment of the risk–benefit ratio of eradicating additional OIF detected by CBCT. The fact that CBCT changed the decision-making for a large proportion of cases is significant, but it does not necessarily mean a better outcome for IE patients (16). This needs to be investigated in clinical trials with a higher level of evidence such as patient outcome efficacy (21). However, due to the rarity of IE, such a study is very difficult or even impossible to perform since it requires recruiting a high number of patients. According to our estimate based on a 5% incidence of IE relapse/recurrence at 1 year (2), a 30% relative reduction in this incidence, a 5% alpha risk, and a 90% power, 7598 patients should be recruited.

The secondary objective of this study was to determine whether practitioners' treatment decisions are influenced by the microbiology of IE. According to the recent position paper of the multidisciplinary task force, a POE in IE patients should be sought regardless of the micro-organism involved (6). The reason given is that all IE patients have a high risk of recurrence, which may be due to micro-organisms different from those involved in the initial IE episode. In four cases of our study, the experts acted without taking into account the causative micro-organism as suggested by this paper. In Case 4 (small but deep decay of the upper left second premolar), the therapeutic approach of the experts was different. A more radical approach (tooth extraction) was recommended when the IE was caused by oral micro-organisms, whereas a conservative endodontic treatment was chosen when a non-oral micro-organism was involved in the IE pathogenesis. This may be explained by the lowest presumed virulence of this situation (ranging 1.2/10 according to the French Society of Oral Surgery), so that the experts adopt a less radical approach and rather opt for a preventive approach than for a curative one. Case 4 highlighted the difficulties in applying the current IE guidelines, since the panelists were aware of this document that was provided with the questionnaire. Their compliance to these guidelines was incomplete probably because the guidelines are non-exhaustive and mainly based on expert opinion.

Our study has unavoidable limitations. First, and in line with the main objective, this study did not directly compare CBCT and OPT. Both images were provided to the experts at the same time. This approach is consistent with clinical practice, as practitioners generally use CBCT as an adjunct to OPT. Second, not all of the CBCT sections were provided to the experts but only a key section highlighting an OIF. To limit this bias, free-text areas were provided, and some experts requested imaging clarifications. This resulted in a rewording of the proposals regarding Case 3 for example. Third, a potential limitation is inherent to the Delphi technique itself: obtaining consensus is not synonymous with a correct answer or judgment (12).

However, this study has several strengths. This is the first Delphi survey to obtain a consensus on OIF detection and management of IE patients, and the first to assess the impact of CBCT on decision-making regarding the OIF in these patients. This process was anonymous (to avoid social pressure and conformity to a dominant point of view) and iterated with feedback (allowing for a change of opinion). The guidelines on conducting and reporting Delphi studies (CREDES) were followed to ensure the quality of the study (12). This multicentric French survey included more than 17 selected experts in the field of OIF management, who fully completed all rounds. The delay between the first and fourth round was shortened (3 months) to prevent memory bias. The participation rate was high in each round (≥85%) showing that the risk of selection bias was low. It even improved as the survey progressed, revealing that the attrition bias commonly found in such studies was avoided. Additionally, experts’ comments were systematically studied and used to reword the initial proposal if necessary.

## Conclusions

In conclusion, using the Delphi procedure, an expert consensus was reached on the decision-making regarding OIF revealed by CBCT in IE patients instead of using only preliminary oral examination coupling with an OPT. CBCT markedly influenced the decision-making concerning OIF by leading to the eradication of more OIF than with conventional OPT. This impact may be clinically relevant because OIF could be potentially responsible for the relapse or recurrence of life-threatening IE. Our findings represent a relevant step toward the development of future studies to further explore the efficacy of CBCT on curative or preventive treatment of IE patients.
